# A Digital Workflow for Interdisciplinary Full-Mouth Rehabilitation: Integration of Orthodontics and Prosthodontics

**DOI:** 10.3390/jcm15197511

**Published:** 2026-09-27

**Authors:** Carlos A. Jurado, Francisco Garcia-Torres, Ma. Guadalupe Velazquez-Verdad, Silvia Rojas-Rueda, Noe de la Cruz, Abdullah Alhussein, Abdullah Almansour, Abdulrahman Alshabib

**Affiliations:** 1 School of Dental Medicine, Ponce Health Sciences University, Ponce 00716, Puerto Rico; 2 Department of Prosthodontics and Implantology, School of Dentistry, University of La Salle Bajio, Leon 37150, Mexico; 3 Orthodontics Private Practice, Leon 37150, Mexico; 4 Kronos Dental Laboratory, Leon 37150, Mexico; 5 Department of Restorative Dental Science, College of Dentistry, King Saud University, Riyadh 11545, Saudi Arabia; aalhussein@ksu.edu.sa; 6 College of Dentistry, Prince Sattam Bin Abdulaziz University, Al-Kharj 11942, Saudi Arabia

**Keywords:** CAD/CAM, case report, occlusal veneers, zirconia

## Abstract

A 60-year-old male patient presented seeking replacement of missing posterior teeth and improvement of his smile. Clinical findings included partial edentulism, bilateral posterior crossbite, secondary caries, defective restorations, anterior and posterior tooth wear, posterior gingival inflammation, supraeruption of the mandibular posterior teeth, and unfavorable inclination of the mandibular posterior teeth. An interdisciplinary treatment plan combining orthodontic, implant, and restorative therapy was selected to improve tooth position, occlusion, restorative space, and esthetics before definitive rehabilitation. Following orthodontic treatment, a digital workflow incorporating intraoral scanning, digital wax-up, restorative space evaluation, and computer-aided design was used to plan a full-mouth rehabilitation. Implant-supported restorations replaced the missing posterior teeth, while the remaining dentition was restored with tooth-supported restorations. Partial-coverage restorations and occlusal veneers were used posteriorly to preserve sound tooth structure, and minimally invasive labial veneers were used anteriorly to improve esthetics. Zirconia was selected because of its favorable mechanical properties and clinical versatility. Cementation was performed under complete dental dam isolation to minimize contamination. This case illustrates how a staged interdisciplinary approach supported by digital planning can facilitate conservative full-mouth rehabilitation and maintain satisfactory functional and esthetic outcomes over a 3-year follow-up.

## 1. Introduction

Complex full-mouth rehabilitation often requires coordinated care among orthodontic, periodontal, implant, and restorative disciplines. Tooth position, periodontal support, occlusal relationships, restorative space, soft-tissue architecture, and implant-site requirements are interdependent; therefore, diagnosis and treatment sequencing should be guided by the anticipated restorative endpoint [1,2,3,4,5,6]. Staged treatment permits disease control and stabilization before orthodontic, surgical, and restorative procedures and allows reassessment between treatment phases [7,8].

Pre-restorative orthodontic therapy can improve tooth position, axial inclination, occlusal relationships, and space distribution before definitive rehabilitation [9,10,11,12]. Correction of crowding, tipping, extrusion, and unfavorable tooth inclinations may reduce the need for excessive tooth preparation and overcontoured restorations while improving esthetic proportions, restorative space, proximal relationships, and load distribution [13,14,15,16]. In partially edentulous patients, orthodontic space redistribution may also establish more favorable conditions for implant placement and subsequent prosthetic rehabilitation.

Digital workflows complement this interdisciplinary approach through the integration of intraoral scanning, digital photography, virtual diagnostic wax-ups, computer-aided design, and computer-aided manufacturing [17,18,19,20]. These technologies allow the treatment team to visualize the restorative endpoint, evaluate restorative space and occlusion, transfer the proposed design to diagnostic mock-ups and provisional restorations, and maintain a common reference throughout treatment.

Despite these advances, clinical reports documenting the complete sequence from pre-restorative orthodontic correction through implant placement, minimally invasive full-mouth rehabilitation, adhesive cementation, and longitudinal evaluation remain limited. Therefore, the aim of this clinical case report was to describe a staged interdisciplinary digital workflow in which orthodontic therapy was used to optimize tooth position and restorative space before implant and restorative treatment, followed by conservative zirconia restorations and a 3-year clinical follow-up. This case report was prepared in accordance with the CARE guidelines. The report emphasizes the integration of treatment sequencing, digital transfer of the restorative plan, material selection, and adhesive procedures in the management of a complex clinical condition.

## 2. Clinical Case Presentation

A 60-year-old male patient presented to the clinic with the chief complaint of wanting to “fix his entire mouth.” He reported discomfort with his bite due to multiple missing teeth and expressed a desire to improve the overall esthetics of his anterior dentition.

His medical history revealed no allergies, he denied taking any medications, and he stated that he was in good health, classifying him as ASA type I. Family and psychosocial histories were noncontributory. A comprehensive clinical, digital, and radiographic evaluation was performed. The patient was diagnosed with partial edentulism, bilateral posterior crossbite, secondary caries, defective restorations, incisal wear of the anterior teeth, occlusal wear of the posterior teeth, gingivitis in both posterior regions, supraeruption of the mandibular posterior teeth, and mesial inclination of the mandibular right second and third molars and the mandibular left third molar (Figure 1).

The patient stated that he did not want any type of removable prosthesis and requested treatment with fixed dental prostheses. He was informed of the complexity of his condition and the need for a multidisciplinary approach involving orthodontics, periodontics, implant dentistry, and restorative dentistry to achieve periodontal health, a stable occlusal scheme, and satisfactory esthetics. The patient understood and accepted the proposed multidisciplinary treatment plan.

The patient was initially referred for a comprehensive periodontal evaluation. Periodontal treatment included plaque and calculus removal, management of gingival inflammation, and oral hygiene instruction. In addition, the mandibular third molars were extracted because of bone loss and Grade II mobility and to create the space required for future implant therapy.

Once periodontal health was established, the patient was referred for orthodontic treatment, which was divided into five phases. Phase I focused on anchorage and transverse control. Bands with buccal tubes and lingual sheaths were cemented to control the transverse dimensions of the dental arches and improve the relationship between the maxillary and mandibular dentitions. Phase II involved segmentation and initial alignment, with passive self-ligating appliances placed (Roth 0.022”, Damon System, Ormco, Brea, CA, USA) on the premolars and canines to initiate controlled tooth movement. During Phase III, orthodontic mechanics were progressively advanced to align and upright the tilted teeth. Phase IV addressed dentoalveolar asymmetry and arch coordination through the placement of full multibracket appliances to optimize the transverse and sagittal interarch relationships. During Phase V, the space required for implant placement was achieved, the orthodontic appliances were removed, and the corrected tooth positions were stabilized with retainers (Figure 2).

During the final stage of orthodontic treatment, before removal of the appliances, an intraoral scan was obtained and a digital wax-up of the proposed restorations was created to confirm that the desired tooth positions had been achieved (Figure 3).

After the conclusion of the two years orthodontic treatment, a digital wax-up (MSOFT, MIS Dental Implants, Misgav, Israel) was subsequently used to plan implant placement in the mandibular left first molar and mandibular right first and second molar regions (Figure 4).

Implants with 4.2 mm diameter by 10 mm length (MIS Implants Technologies, Bar-Lev, Israel) were placed using previously designed surgical guides, during implant placement, the implant stability quotient (ISQ) values obtained for the three implants ranged from 81 to 86 and were considered indicative of adequate primary stability. After four months of healing, a new intraoral scan was obtained and an updated digital wax-up was created (Figure 5).

Printed models (Phrozen Sonic Mighty 4K Resin 3D Printer, Phrozen Technology, Hsinchu City, Taiwan) were fabricated from the digital wax-up, and silicone matrices (Elite P&P, Zhermack, Badia Polesine, Italy) were produced to create an intraoral diagnostic mock-up (Structur 3, VOCO Dental, Cuxhaven, Germany). The occlusion and excursive movements were evaluated with the mock-up in place (Figure 6).

The patient was satisfied with the proposed outcome and requested continuation of treatment. Teeth (14, 15, 16, 24, 25, 26) with defective restorations and/or caries were treated using a minimally invasive approach under dental dam isolation (MDC Dental, Zapopan, Mexico). Core buildups were performed using composite resin (GC EverX Posterior, GC, Tokyo, Japan), and interim restorations fabricated from provisional material (Structure 2, VOCO, Cuxhaven, Germany) were placed at a new vertical dimension of occlusion, increased by 3 mm (Figure 7).

The full-mouth interim restorations were evaluated in maximum intercuspation and during excursive movements (Figure 8).

The patient wore the interim restorations for three months and reported that they were comfortable and functioned satisfactorily. The previous diagnostic digital wax-up used for the provisional restorations was also used as a reference to fabricate the final digital design of the restorations. He subsequently requested continuation of treatment. Retraction cord (#00 Ultrapak, Ultradent, South Jorda, UT, USA) was placed along the gingival margins. Final photographs were obtained for shade selection, and a definitive digital impression (Medit i700, Medit, Seoul, Republic of Korea) was acquired for the fabrication of the tooth- and implant-supported restorations. The definitive intraoral scan was performed in sections. First, the anterior provisional restorations were removed and the anterior region was scanned, while the posterior provisionals remained in place to preserve the established interarch space. The anterior provisionals were then repositioned, and the posterior provisional restorations on both the right and left sides were removed and scanned while the anterior provisionals maintained the established spatial relationship. Finally, the anterior and posterior scans were stitched together to generate a single digital scan representing the complete restorative space required for fabrication of the definitive restorations (Figure 9).

To achieve a natural appearance, shade A2 was selected for the restorations. Because the teeth did not exhibit any intrinsic discoloration, no additional masking of the underlying tooth structure was required by the dental technician. The definitive zirconia restorations (Prettau 3 Dispersive, Zirkonzahn, Gais, Italy) were fabricated and evaluated on printed models generated from the final digital impression (Figure 10).

Prior to treatment of the ceramic restorations and tooth surfaces, the anterior interim restorations were removed. The definitive restorations were then tried in without cement to evaluate marginal adaptation and shade. Both the clinician and the patient assessed the esthetic outcome, and the patient expressed satisfaction with the restorations and agreed to proceed with final cementation.

First, the restorations were airborne-particle abraded with 50-μm aluminum oxide particles (Cobra Aluminum Oxide, Renfert GmbH, Hilzingen, Germany) at a pressure of 2 bar. They were then cleaned with a zirconia cleaning paste (Zirclean, Bisco Inc., Schaumburg, IL, USA), followed by ultrasonic cleaning in water for 5 min. A ceramic primer (Clearfil Ceramic Primer Plus, Kuraray Noritake Dental Inc., Tokyo, Japan) was subsequently applied. The teeth were airborne-particle abraded with 29-μm aluminum oxide particles (AquaCare, Velopex International, London, UK) and then etched with 37% phosphoric acid (Ultra-Etch, Ultradent, South Jordan, UT, USA) for 20 s. An adhesive (All-Bond Universal, Bisco Inc., Schaumburg, IL, USA) was applied and air-thinned. Finally, the definitive restorations were cemented using resin cement (Panavia V5, Kuraray Noritake, Tokyo, Japan). The implant-supported zirconia restorations were bonded extraorally to titanium bases and subsequently screw retained to the implants. The intaglio surface of the zirconia and the titanium-base bonding surface were conditioned according to the manufacturers’ recommendations, and the restorations were bonded to the titanium bases using a Multilink Hybrid Abutment (Ivoclar, Schaan, Linchenstein). The prosthetic screws were tightened to the manufacturer-recommended torque of 30 Ncm, and the screw-access channels were subsequently sealed with PTFE tape and composite resin (Filtek Supreme, 3M, St Paul, MN, USA). The occlusion was evaluated, the restorations were polished using a ceramic polishing system (Eve Diacera, Keltern, Germany), and a final panoramic radiograph was obtained (Figure 11).

The patient was satisfied with the treatment outcome. Oral hygiene instructions were provided, and an occlusal splint was delivered. The patient was instructed to wear the guard at night and return every six months for dental prophylaxis and evaluation of the restorations.

At the three-year follow-up, a comprehensive periodontal assessment, including periodontal probing, demonstrated healthy periodontal conditions around the teeth and implants. Occlusion was also evaluated and remained stable, with satisfactory functional contacts. The restorations were in good clinical condition, with acceptable marginal integrity, esthetics, and overall function. Moreover, the patient reported that the treatment met his functional and esthetic expectations and remained satisfied with the outcome at the 3-year follow-up. No adverse or unanticipated events, including restoration fracture, debonding, or implant-related complications, were observed during the 3-year follow-up (Figure 12).

## 3. Results

Pretreatment clinical findings included partial edentulism, bilateral posterior crossbite, secondary caries, defective restorations, anterior and posterior tooth wear, posterior gingival inflammation, supraeruption of the mandibular posterior teeth, and mesial inclination of the mandibular posterior teeth. Following two years of orthodontic treatment, tooth alignment and interarch relationships were improved, and adequate restorative and implant space was established for the planned rehabilitation.

Three implants measuring 4.2 mm × 10 mm were placed in the mandibular left first molar and mandibular right first and second molar regions. Primary implant stability values ranged from 81 to 86 ISQ. The definitive restorative phase was performed at a vertical dimension of occlusion increased by 3 mm, which had been evaluated with interim restorations for 3 months before definitive treatment. The completed rehabilitation restored posterior support, tooth form, proximal relationships, and the planned occlusal scheme.

At the 3-year follow-up, periodontal probing demonstrated healthy periodontal and peri-implant conditions. Occlusal evaluation showed maintenance of stable functional contacts, and the restorations demonstrated acceptable marginal integrity, esthetics, and function. The patient remained satisfied with the treatment outcome.

## 4. Discussion

The present case report describes a comprehensive interdisciplinary approach developed to address the patient’s functional, biologic, and esthetic concerns. In older patients, tooth loss may lead to progressive changes such as mesial or distal tilting of adjacent teeth, drifting, extrusion of unopposed teeth, loss of arch continuity, reduction in available prosthetic space, and alteration of the occlusal plane. These changes can compromise implant positioning, restorative contours, interarch relationships, and the distribution of occlusal forces. Therefore, treatment required coordinated orthodontic therapy to upright tilted teeth, redistribute and recreate appropriate edentulous spaces, correct unfavorable tooth positions, and establish a more ideal occlusal plane before implant placement and definitive rehabilitation. Each phase was planned according to the anticipated restorative outcome so that tooth position, available restorative space, implant location, occlusal relationships, and esthetic parameters could be evaluated before irreversible treatment was initiated. Digital technologies were incorporated throughout the workflow, including intraoral scanning, virtual treatment planning, digital diagnostic wax-ups, computer-aided restoration design, and fabrication of milled definitive restorations. The digital wax-up was used not only to visualize the proposed outcome but also to guide the diagnostic mock-up, orthodontic objectives, tooth preparation, provisionalization, and fabrication of the definitive prostheses.

The restorative material used for the dental treatment was a multilayer zirconia. The zirconia materials currently available to clinicians include 3Y, 4Y, 5Y, and multilayer zirconia. A key advantage of multilayer zirconia is that it combines layers with different yttria concentrations and, consequently, different mechanical and optical properties. The 5Y layer, which provides the highest translucency, is typically positioned in the incisal region, while the 3Y or 4Y layers are located in the middle and gingival regions. This configuration is intended to provide greater strength in the body of the restoration while maintaining enhanced translucency and esthetics in the incisal portion. A detailed description of the current types of zirconia is provided in Table 1 [21,22,23,24,25].

A minimally invasive preparation strategy was selected to preserve as much sound tooth structure as possible. In the posterior region, partial-coverage restorations were used instead of conventional full-coverage crowns whenever the remaining tooth structure and restorative space permitted. In the anterior region, zirconia veneers were selected to improve tooth form, proportion, alignment, and color while minimizing preparation depth. Following try-in and verification of marginal adaptation, proximal contacts, esthetics, and occlusion, the restorations were adhesively cemented under complete rubber dam isolation. This approach was intended to prevent contamination of the bonding surfaces by saliva, blood, or crevicular fluid and to optimize the consistency and effectiveness of the adhesive cementation protocol.

Orthodontic therapy is a conservative treatment modality that can be used in a wide range of clinical situations, including correction of malocclusion, alignment of crowded or rotated teeth, uprighting of tilted teeth, and redistribution of spaces according to restorative and implant-related requirements [26,27]. In patients requiring extensive rehabilitation, orthodontic treatment should not be viewed as an isolated preliminary procedure, but rather as an integral component of the definitive restorative plan. When performed before full-mouth rehabilitation, orthodontic therapy can improve the position and axial inclination of the teeth, establish more favorable interarch relationships, and create the restorative space necessary for conservative and appropriately contoured restorations.

The benefits of pre-restorative orthodontic treatment extend beyond esthetic alignment. Uprighting severely tilted teeth may improve their periodontal maintainability, redistribute occlusal forces more favorably, and, in selected situations, prevent unnecessary extraction. Correction of the occlusal plane and tooth inclination may also reduce the need for aggressive tooth reduction or overcontoured restorations intended to compensate for unfavorable tooth positions. Similarly, closing excessive spaces can facilitate the fabrication of restorations with more natural dimensions, improved proximal contacts, and appropriate emergence profiles. Conversely, controlled opening or redistribution of space may provide the mesiodistal width required for implant placement and allow the implant-supported restoration to reproduce more ideal tooth proportions [28,29]. Proper root positioning is particularly important in implant sites because adequate interradicular space is required not only for implant placement but also for preservation of the supporting bone and development of a favorable emergence profile.

Orthodontic movement may also improve the relationship between the restorative margins and the surrounding periodontal tissues. By repositioning teeth into more favorable locations, the restorative dentist may be able to preserve enamel, reduce preparation depth, and create restorations that are easier for the patient to maintain. Furthermore, orthodontic correction can assist in the development of appropriate anterior guidance, posterior disclusion, and occlusal load distribution. These factors are particularly relevant in full-mouth rehabilitation, where multiple restorations must function as a coordinated occlusal system. Several clinical studies have reported favorable outcomes when orthodontic therapy is integrated with comprehensive restorative treatment; the principal findings of these studies are summarized in Table 2 [30,31,32,33,34,35,36,37,38,39].

The restorative workflow used in the present clinical report was fully digital. It included an initial diagnostic intraoral scan, digital diagnostic wax-ups, fabrication of a diagnostic mock-up, computer-aided design of the definitive restorations, and production of the final milled prostheses. The intraoral scan provided a digital representation of the dentition that could be evaluated together with the clinical photographs and restorative plan. The digital wax-up allowed the clinicians and dental technician to assess tooth dimensions, occlusal relationships, restorative thickness, incisal-edge position, and esthetic integration before tooth preparation. It also served as a reference for transferring the approved design to the diagnostic mock-up and definitive restorations.

Digital workflows have demonstrated clinically acceptable results for the fabrication of inlays, crowns, and tooth-supported fixed partial prostheses [40,41]. Although the accuracy of any digital workflow depends on the scanner, scanning strategy, software, restorative material, milling system, and clinical conditions, digital impressions may eliminate several variables associated with conventional elastomeric impressions, including tray distortion, material deformation, tearing, voids, and dimensional changes during storage or transportation. Digital records can also be immediately reviewed, and areas with incomplete data can be rescanned without repeating the entire impression procedure.

A systematic review evaluating the clinical performance of CAD/CAM tooth-supported restorations reported that these restorations appear to perform similarly to restorations fabricated using conventional techniques [42]. This finding supports the use of digital manufacturing when the clinical indication, preparation design, restorative material, and adhesive protocol are appropriately selected. In addition, laboratory evidence has indicated that CAD/CAM fabrication may require significantly less production time than conventional laboratory workflows [43]. The reduction in manufacturing time may be associated with the elimination of several conventional steps, including impression pouring, die trimming, waxing, investing, and casting or pressing, depending on the restorative system used.

Patients have also reported a preference for digital workflows, particularly digital impressions, compared with conventional impression techniques [44]. This preference may be related to reduced gagging, improved comfort, shorter chairside procedures, and the ability to visualize the scanned dentition immediately. Digital technology may also facilitate patient communication by allowing the proposed treatment outcome to be presented before definitive procedures are performed. In extensive rehabilitations, this visual communication can be particularly valuable because patients may have difficulty understanding the effects of changes in tooth length, tooth position, occlusal plane, or restorative contours based solely on verbal explanations.

The present full-mouth rehabilitation incorporated posterior partial-coverage restorations as part of a minimally invasive treatment strategy. The primary objective was to restore form, function, and occlusal stability while preserving sound enamel and dentin. Compared with conventional full-coverage crowns, partial restorations may require less circumferential and axial tooth reduction, thereby preserving additional tooth structure and potentially decreasing the biological risks associated with extensive preparation. Adhesive ceramic restorations can also use the remaining enamel for bonding, which is particularly advantageous when adequate isolation and appropriate adhesive procedures can be achieved.

Partial-coverage restorations have demonstrated favorable mechanical behavior in several investigations. A recent laboratory study evaluated the effect of preparation design on the fracture behavior of lithium disilicate ceramic occlusal veneers placed on maxillary premolars. Four conservative designs were investigated: an occlusal veneer (O) and three partial-coverage configurations designated OF, POF, and POFP. These designs were compared with a conventional full-coverage crown (C). After adhesive cementation to natural teeth, the specimens were subjected to 20,000 cycles of compressive loading and subsequently loaded until fracture. The occlusal veneer and partial-restoration groups demonstrated higher fracture-resistance values than the conventional full-coverage crown group. The mean fracture-resistance values were 2153 N for the occlusal veneer design, 1998 N for the OF design, 1596 N for the POF design, and 1540 N for the POFP design, compared with 1428 N for the conventional crown. The authors concluded that conservative partial-coverage restorations may represent a viable alternative to full-coverage crowns for the rehabilitation of severely worn teeth [45]. These fracture-resistance values were obtained from an in vitro study using lithium disilicate restorations and therefore should be interpreted as general supporting evidence for conservative preparation designs rather than as mechanical data directly applicable to the Prettau 3 Dispersive zirconia used in the present case. Moreover, these findings also suggest that preservation of tooth structure does not necessarily compromise mechanical performance when the preparation design, selection of the restorative material, adhesive protocol, and occlusal conditions are properly controlled.

Additional support for conservative posterior restorations has been provided by a systematic review that evaluated the clinical outcomes of occlusal veneers and the influence of material-related parameters on their behavior and survival. The review included studies identified through major electronic databases, including PubMed, BioMed Central, Cochrane, and Scopus. The available experimental evidence supported the use of thin and ultrathin occlusal veneers as conservative restorative options [46]. However, the clinical success of these restorations remains dependent on appropriate case selection, adequate bonding substrate, restorative thickness, ceramic selection, preparation geometry, occlusal loading, and control of parafunctional activity.

In the present case, occlusal veneers were selected for the posterior region to preserve tooth structure while reestablishing occlusal anatomy and function. Nevertheless, minimally invasive restorations should not be interpreted as being free of mechanical risk. Thin ceramic restorations may be vulnerable to excessive or poorly distributed occlusal forces, particularly in patients with bruxism, clenching, or other parafunctional habits. For this reason, an occlusal guard was delivered after completion of treatment to protect the restorations from excessive functional and parafunctional loading. The patient was instructed regarding its use and maintenance. A strict 6-month recall schedule was also recommended. Periodic evaluations are important for monitoring marginal integrity, occlusal contacts, ceramic surface condition, periodontal health, adhesive interfaces, and the fit and condition of the occlusal guard. These appointments also provide an opportunity to identify changes in the patient’s occlusion, parafunctional behavior, or oral-hygiene practices before they contribute to restoration fracture, debonding, wear, or periodontal complications. Long-term maintenance is particularly important in full-mouth rehabilitations because changes affecting one restoration may influence the function and loading of the remaining prostheses.

All definitive tooth-supported restorations were adhesively cemented under complete rubber dam isolation to reduce contamination and improve control of tooth and zirconia surface conditioning [47,48]. Clinical evidence indicates that rubber dam isolation may improve enamel bond strength [49], whereas contamination can adversely affect adhesion to tooth and ceramic substrates [50,51]. Given the number of restorations cemented in this case, complete isolation was used to facilitate consistent execution of the adhesive protocol and minimize contamination.

Complete isolation also improves visualization of preparation margins and facilitates controlled application of etchants, primers, adhesives, and resin cement. It allows excess cement to be removed more precisely and reduces the possibility of moisture affecting the cementation procedure. In addition, isolation may help protect the adjacent teeth and soft tissues during surface conditioning and adhesive application. These advantages are especially important when multiple restorations are cemented during the same appointment and consistent execution of the bonding protocol is required. The rubber dam also contributes to patient safety by protecting the airway and reducing the possibility of accidental ingestion or aspiration of restorative materials, instruments, try-in pastes, or fragments of ceramic [52]. This protective function is particularly relevant during adhesive cementation, when small components and instruments are frequently used. Therefore, the use of complete rubber dam isolation in the present case was intended to support both the biological and mechanical success of the restorations while improving procedural control and patient safety.

A challenge for clinicians is the initial investment required to acquire the equipment necessary for a digital workflow, as well as the associated learning curve. However, the implementation of new technologies can enable faster fabrication of restorations, potentially allowing clinicians to treat more patients and improve practice efficiency and financial performance [53,54,55]. Moreover, studies have shown that patients may prefer digital workflows over conventional methods; therefore, offering advanced digital technologies may also enhance the patient experience and help attract new patients to the practice [56].

A potential limitation of zirconia restorations is the predictability of zirconia-to-enamel bonding. Unlike silica-based ceramics, zirconia cannot be effectively etched with hydrofluoric acid and therefore requires appropriate surface conditioning, such as airborne-particle abrasion and the use of MDP-containing primers or resin cements. Although enamel bonding is generally reliable following phosphoric acid etching, contamination of the zirconia surface, inadequate surface treatment, or insufficient isolation may reduce bond strength [57,58]. Therefore, strict adherence to the recommended bonding protocol is essential to minimize the risk of debonding and improve the long-term clinical performance of zirconia restorations. In the present clinical report, all bonding procedures were performed under complete isolation to minimize contamination and allow the clinician to carefully follow each step of the adhesive protocol.

In the present case, Prettau 3 Dispersive was selected because its multilayer gradient is designed to combine favorable mechanical properties with increased translucency, providing a balance between strength and esthetics for an extensive monolithic rehabilitation [21,22,23,24,25]. This material selection was complemented by airborne-particle abrasion, zirconia cleaning, ceramic priming, resin cementation, and complete rubber dam isolation to optimize the adhesive interface. At the 3-year evaluation, the maintenance of marginal integrity, occlusal stability, esthetics, and overall function was consistent with the intended rationale for this material and cementation protocol; however, the outcome of a single clinical case cannot establish the superiority of this zirconia system or bonding strategy.

Limitations of the present clinical case include the use of only one type of zirconia ceramic. Future clinical studies should evaluate different zirconia formulations to assess their long-term intraoral performance. Another limitation is that the treatment was provided by dental specialists with substantial experience in managing complex cases; therefore, less experienced clinicians should exercise caution when undertaking similarly complex treatment. Finally, future clinical evaluations should compare different resin cement systems to determine their long-term clinical performance and bonding success. In addition, this report describes a single patient without a comparison group, and the clinical follow-up relied primarily on routine clinical assessment rather than standardized quantitative outcome measures. Therefore, the favorable 3-year findings should be interpreted descriptively and cannot demonstrate the superiority of the material, digital workflow, or interdisciplinary treatment approach over alternative treatment strategies.

## 5. Conclusions

The present clinical report demonstrates the value of a staged interdisciplinary approach in which orthodontic therapy was used before full-mouth rehabilitation to optimize tooth position, restorative space, and implant-site conditions. The subsequent restorative phase incorporated implant- and tooth-supported restorations through a digital workflow that included diagnostic scanning, digital wax-up, computer-aided design, and fabrication of milled restorations. In the posterior region, partial-coverage restorations and occlusal veneers were selected to preserve sound tooth structure whenever possible. Clinically, this case highlights the importance of establishing appropriate tooth position before definitive rehabilitation, using digital planning to improve communication and restorative predictability, and maintaining strict adhesive protocols under complete dental dam isolation to minimize contamination. At the 3-year follow-up, the restorations maintained satisfactory fit, occlusal stability, and esthetic integration. These findings support the clinical relevance of combining orthodontic, implant, and minimally invasive restorative strategies in the management of complex full-mouth rehabilitation cases.

## Figures and Tables

**Figure 1:**
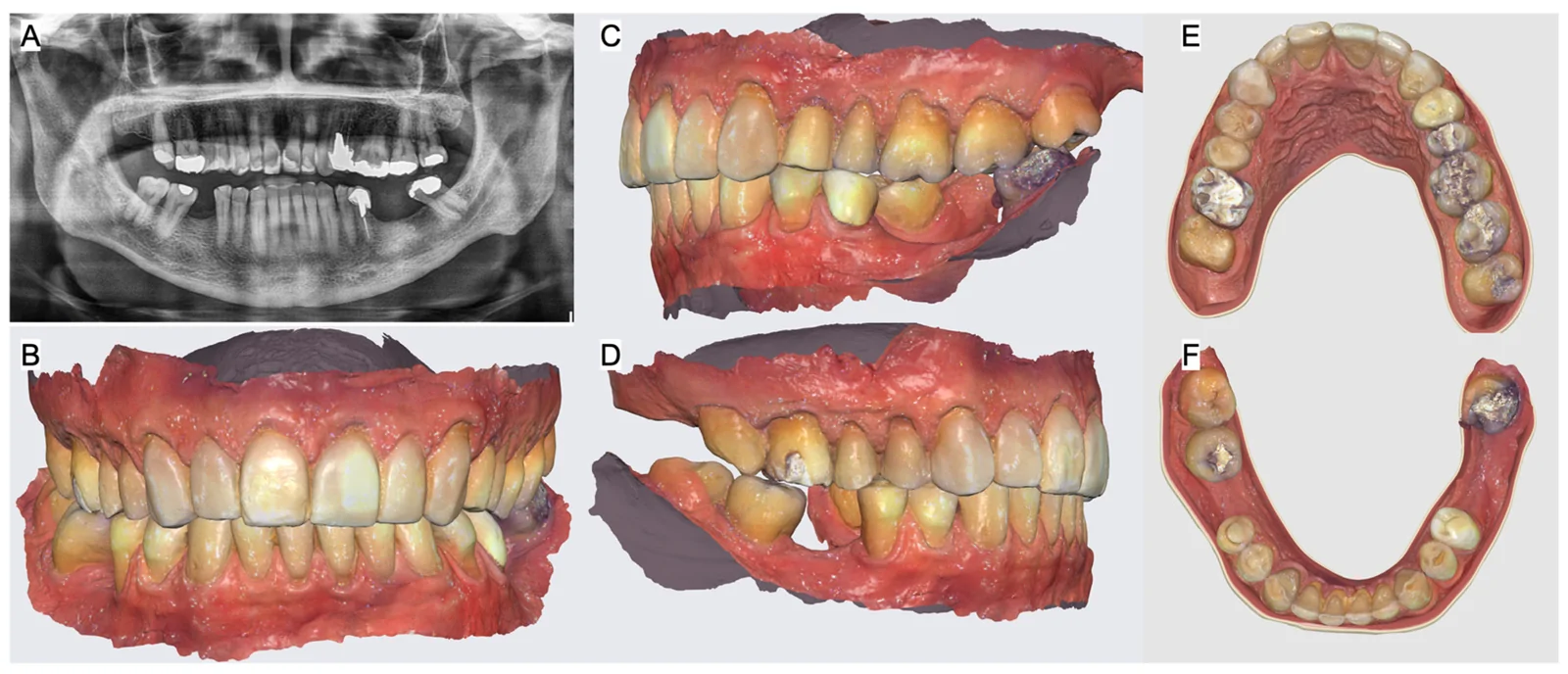
Initial situation. (**A**) Panoramic radiograph, (**B**) frontal, (**C**) left, (**D**) right, (**E**) maxillary and (**F**) mandibular occlusal scan views.

**Figure 2:**
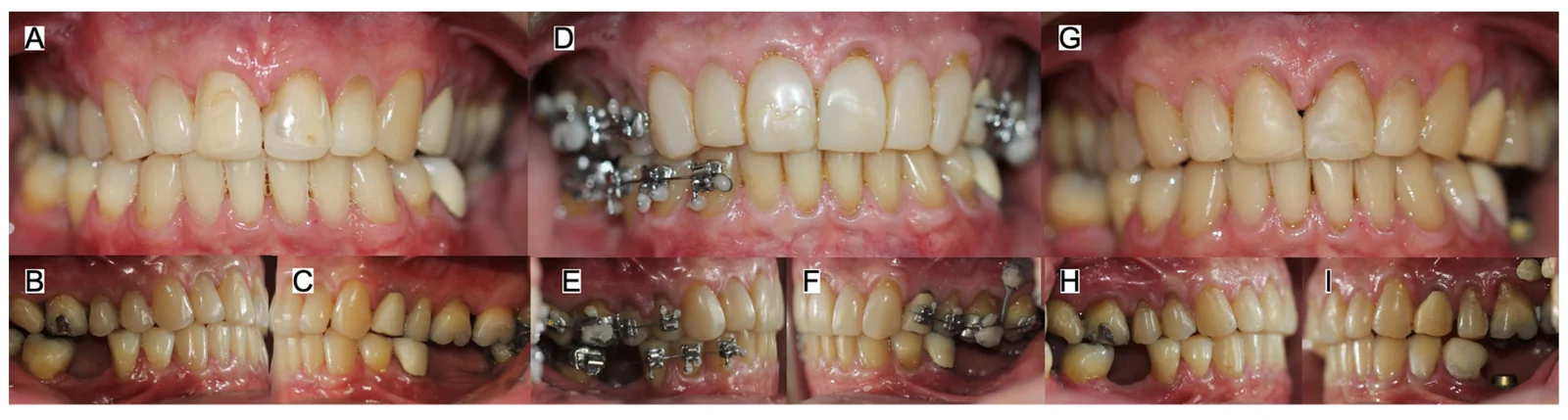
Orthodontic therapy. Initial (**A**) frontal, (**B**) right and (**C**) left side views, during the treatment (**D**) frontal, (**E**) right and (**F**) left side views with orthodontic appliances, and final (**G**) frontal, (**H**) right and (**I**) left side views without orthodontic appliances.

**Figure 3:**
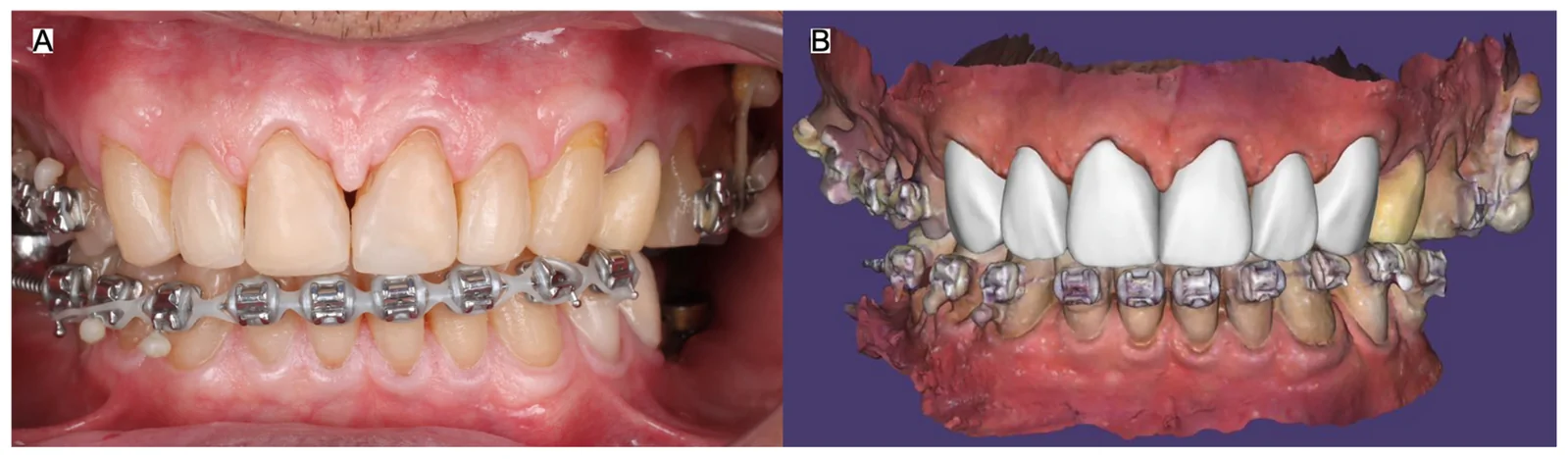
Digital esthetic assessment before finishing orthodontics. (**A**) With orthodontic therapy and (**B**) digital design.

**Figure 4:**
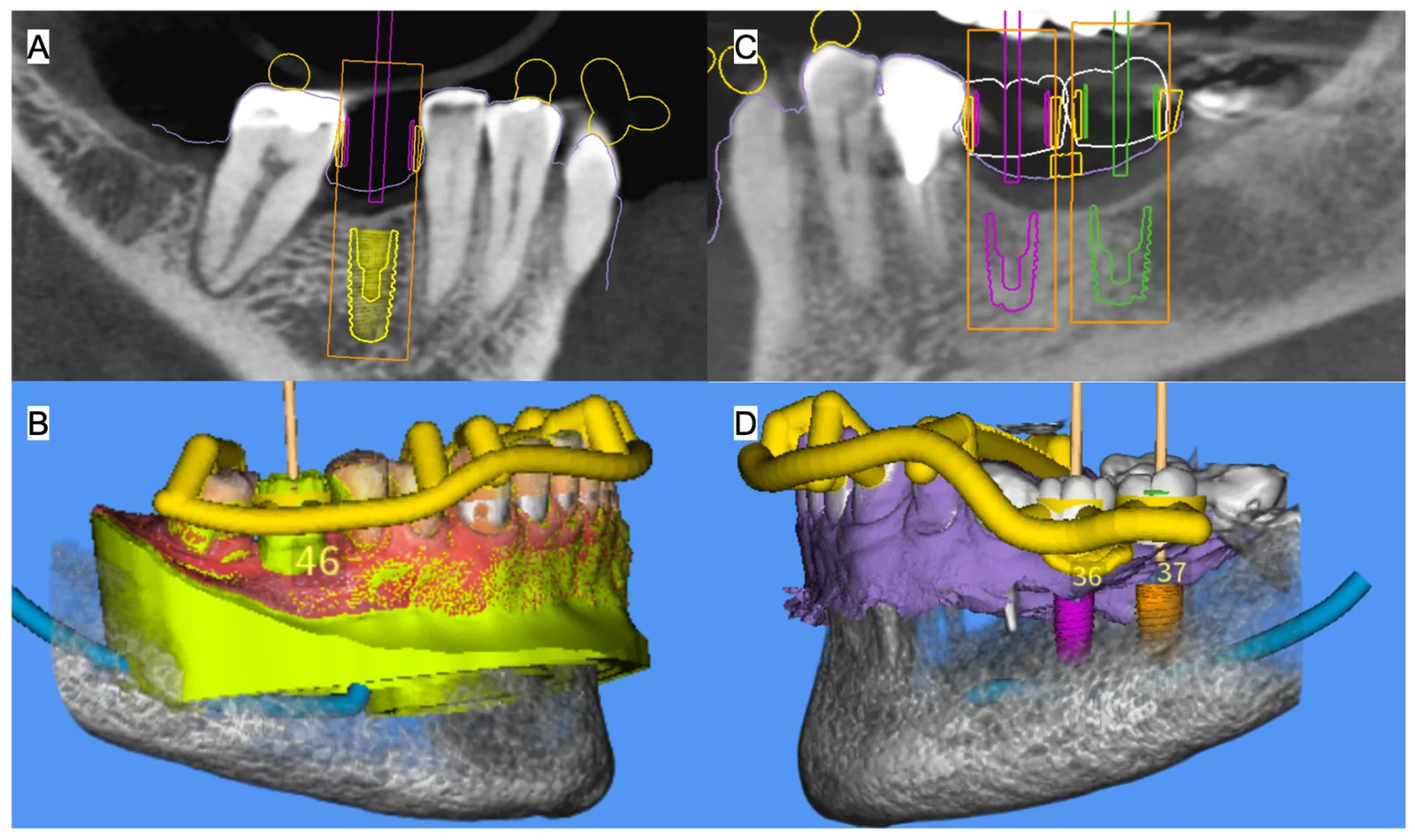
Digital implant planning. (**A**) Implant planning and (**B**) surgical guide design for the mandibular left first molar region; (**C**) implant planning and (**D**) surgical guide design for the mandibular right first and second molar regions.

**Figure 5:**
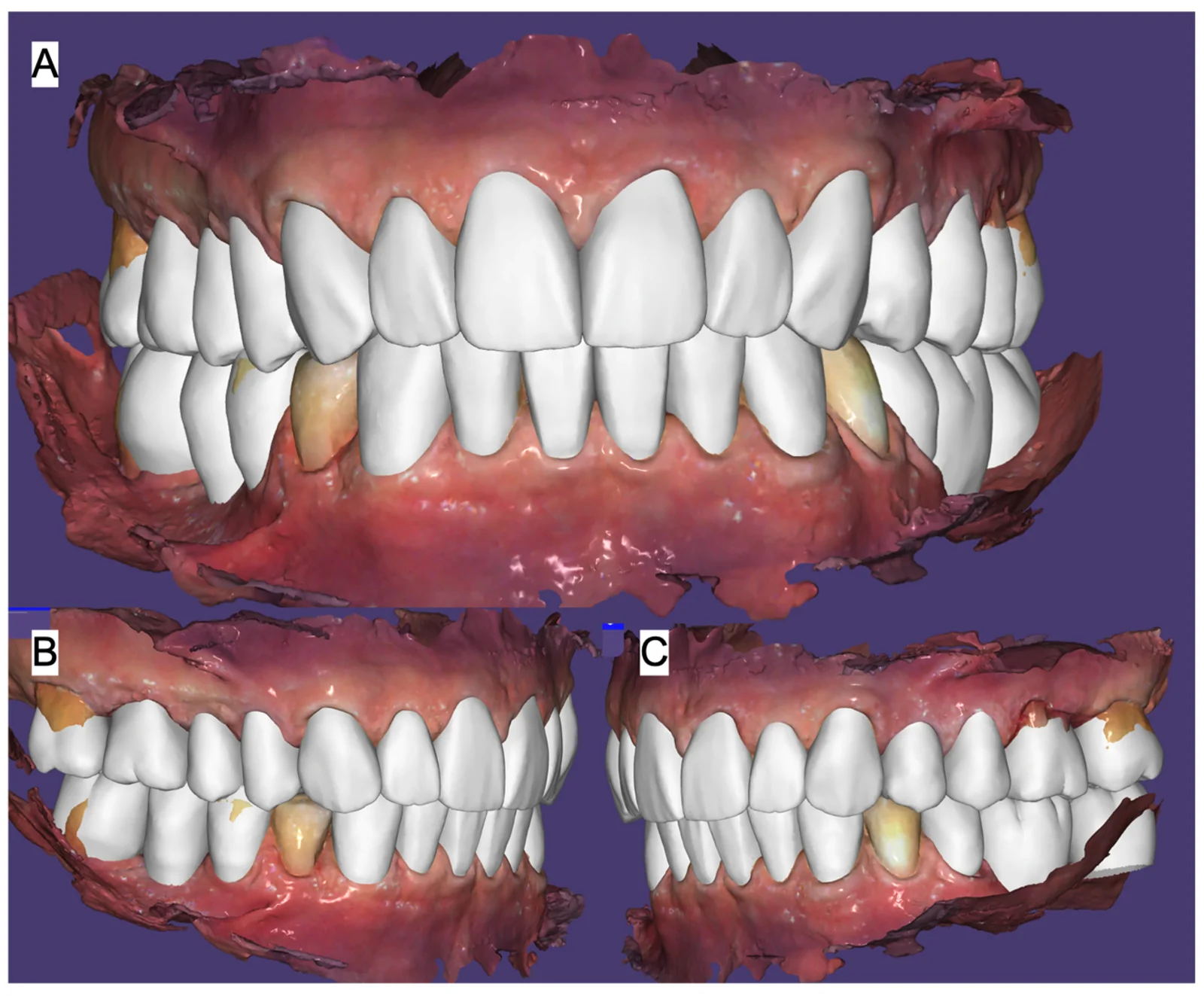
Digital planning for mock-up. (**A**) Frontal, (**B**) left and (**C**) right side view of the digital mock-up in occlusion.

**Figure 6:**
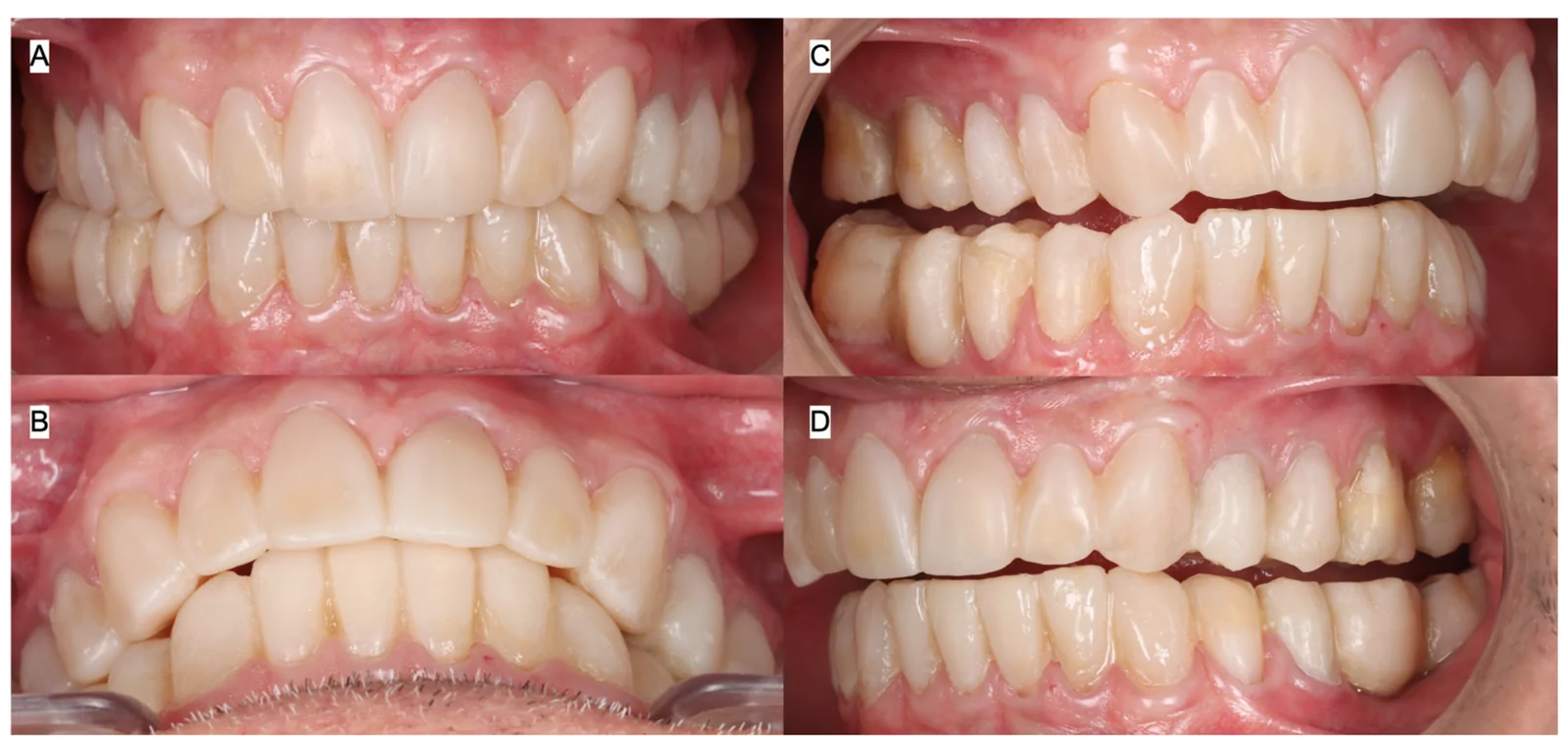
Diagnostic mock-up. (**A**) Frontal, (**B**) incisal contacts, (**C**) right and (**D**) left canine guidance.

**Figure 7:**
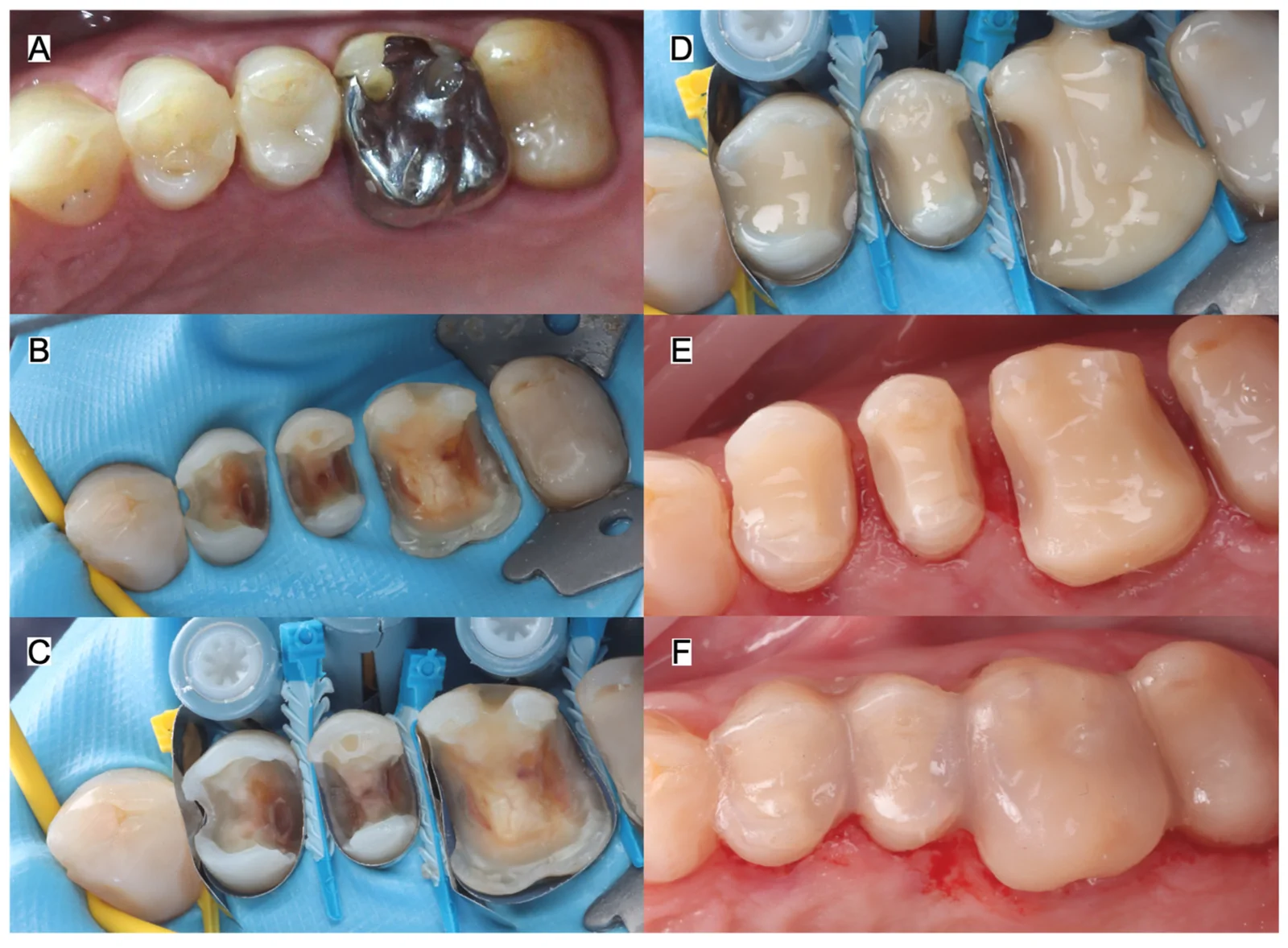
Core build-up prior final restorations. (**A**) Initial situation, (**B**) previous defective restorations removed, (**C**) matrix guides placed, (**D**) composite core-build up placement, (**E**) tooth preparations and (**F**) interim restorations.

**Figure 8:**
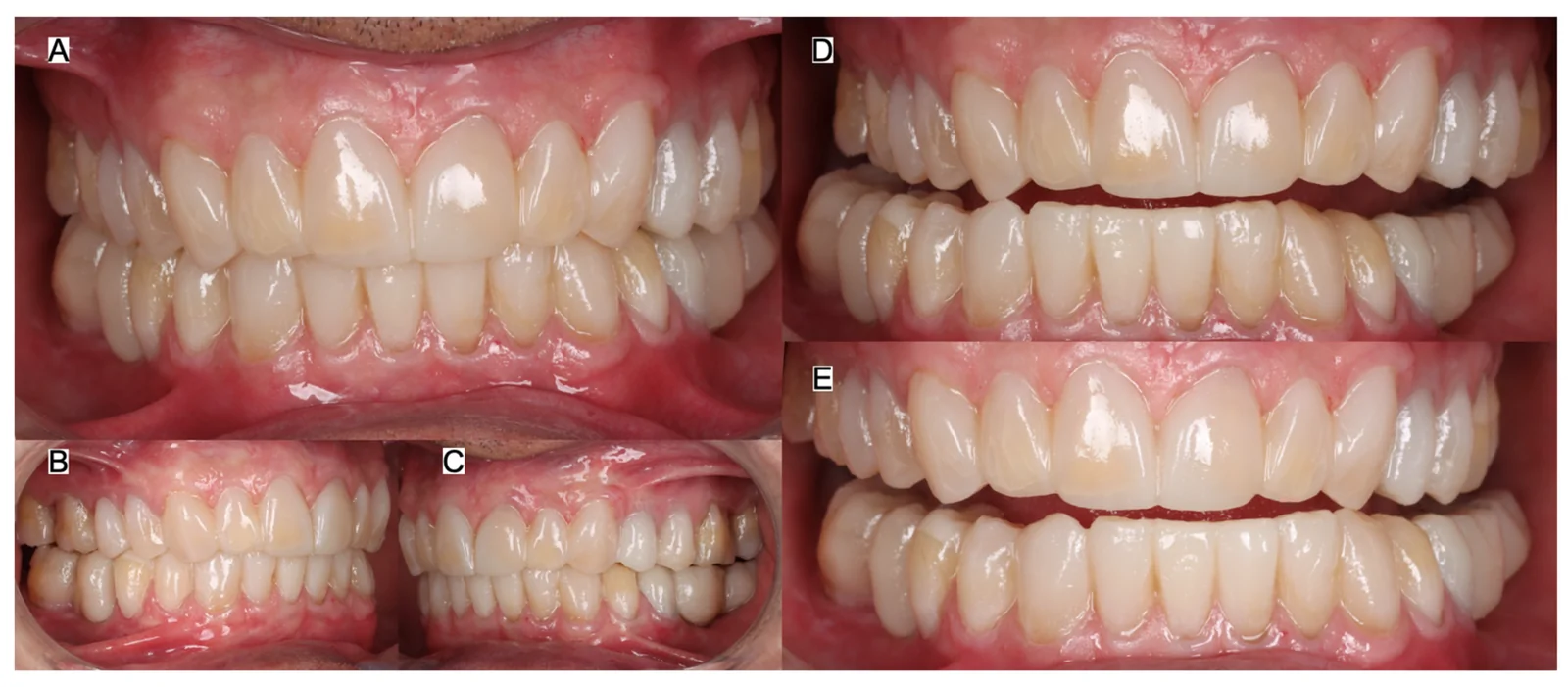
Interim restorations. (**A**) Frontal, (**B**) right and (**C**) left contacts in occlusion, (**D**) right and (**E**) left canine guidance.

**Figure 9:**
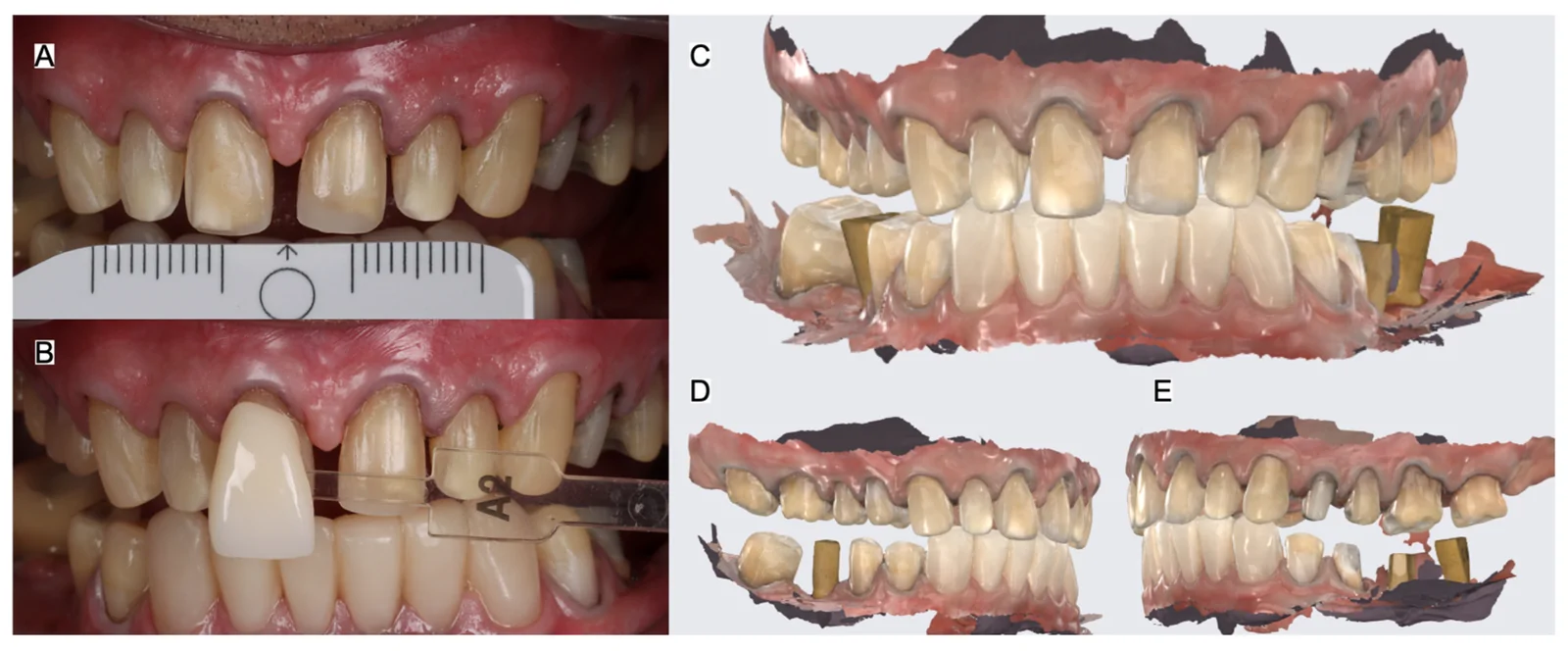
Final shade and digital impression. Photographs with a (**A**) photo calibration card and with (**B**) shade tab; final digital scan (**C**) frontal, (**D**) right and (**E**) left side views.

**Figure 10:**
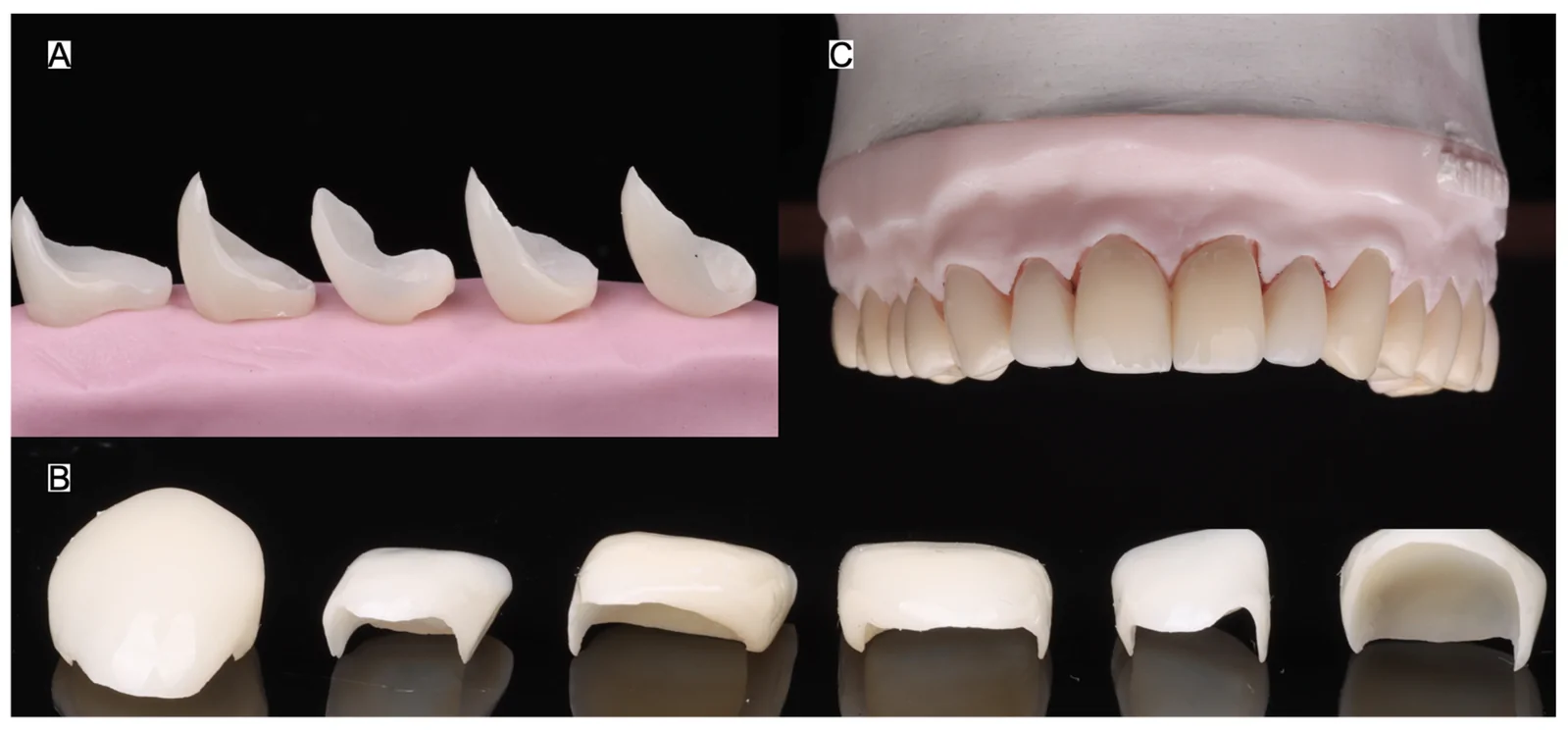
Final restorations. (**A**) Partial posterior restorations, (**B**) frontal anterior restorations and (**C**) all maxillary restorations in a printed model.

**Figure 11:**
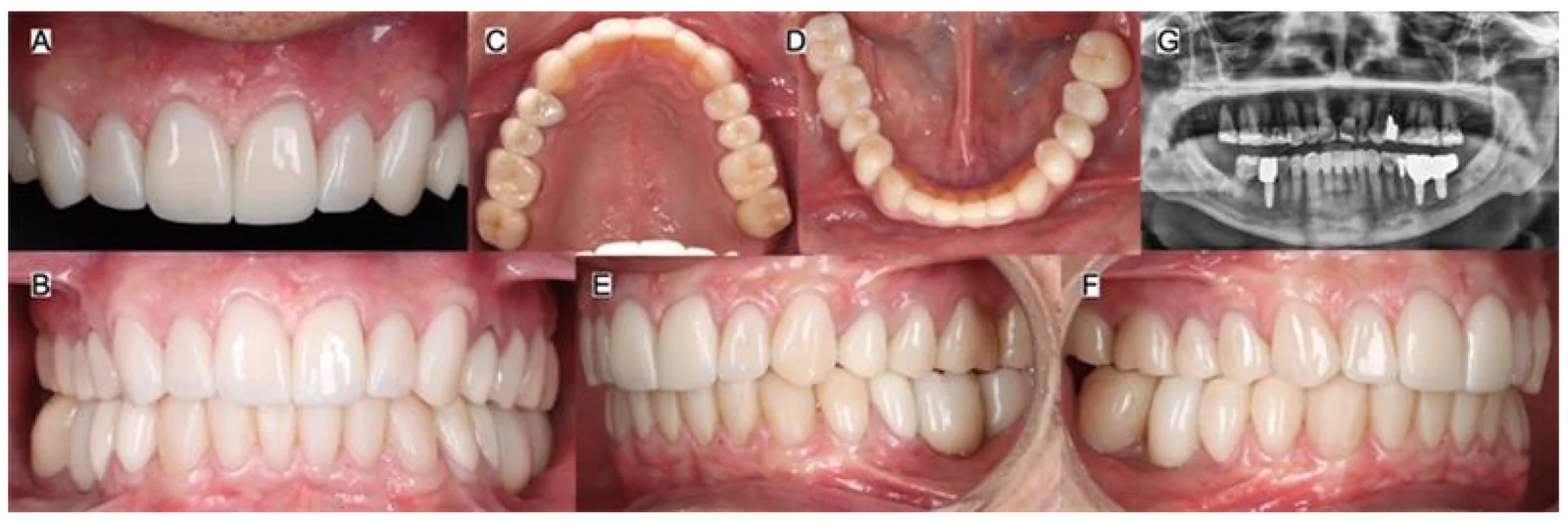
Final restorations placed. (**A**) Anterior frontal, (**B**) all in occlusion, (**C**) maxillary and (**D**) mandibular occlusal, (**E**) left and (**F**) right side views, and (**G**) final panoramic radiograph.

**Figure 12:**
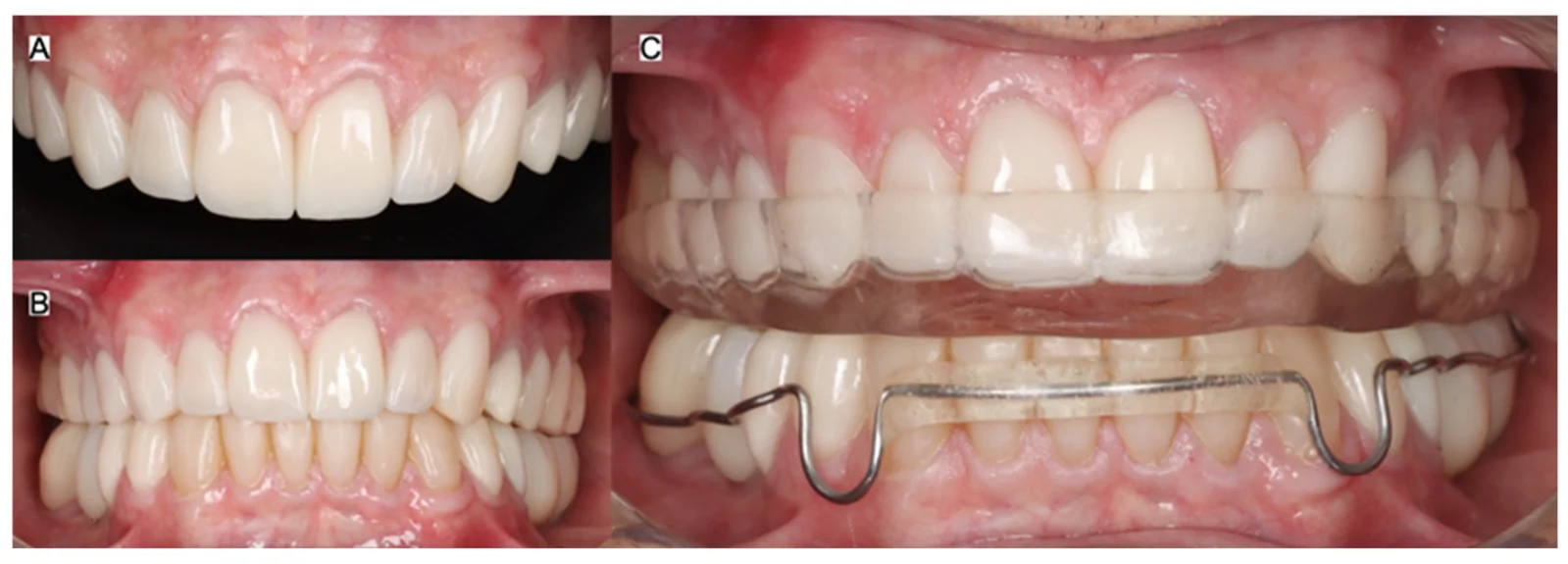
Three years follow-up. (**A**) Anterior teeth, (**B**) all in occlusion and (**C**) wearing night guard and orthodontic retainer.

**Table 1:** Dental zirconia types, composition and clinical uses [21,22,23,24,25].

Type	Chemical Composition	Uses in Dentistry	Flexural Strength
3Y Zirconia	3Y-TZP: ZrO_2_ stabilized with approximately 3 mol% Y_2_O_3_; predominantly tetragonal phase	Posterior crowns, long-span fixed dental prostheses, implant-supported restorations, and frameworks. Highest strength, lower translucency.	900–1200 MPa
4Y Zirconia	4Y-PSZ: ZrO_2_ stabilized with approximately 4 mol% Y_2_O_3_; increased cubic-phase content	Anterior and posterior monolithic crowns and short- to medium-span fixed dental prostheses. Balance between strength and translucency.	800–1100 MPa
5Y Zirconia	5Y-PSZ: ZrO_2_ stabilized with approximately 5 mol% Y_2_O_3_; higher cubic-phase content	Highly esthetic anterior crowns, veneers, and selected posterior restorations. Highest translucency, lower strength.	500–800 MPa
Multilayered Zirconia	Multiple layers with color/ translucency gradients; some systems also incorporate different yttria concentrations (e.g., 3Y, 4Y, and/or 5Y)	Monolithic anterior and posterior restorations requiring a natural cervical-to-incisal esthetic gradient; applications depend on the zirconia composition within each layer.	Incisal/Occlusal: 500–600 MPa Cervical: 900–1100 MPa

**Table 2:** Summary of clinical studies combining comprehensive care combining orthodontic and restorative dentistry.

Study and Authors	Methodology	Results	Follow-Up
Orthodontic-restorative case report. Giacobbo et al. [30], 2019.	A 38-year-old patient with Class I malocclusion, posterior crossbite, and tooth wear was treated with fixed orthodontics, a mini-expandex-type expander, vertical elastics, and restorative rehabilitation.	Orthodontic correction created space and occlusal stability for conservative restoration of the worn teeth.	37 months
Interdisciplinary adult orthodontic clinical article. Schneider et al. [31], 2024.	The article presents complex adult orthodontic cases managed through coordinated orthodontic, restorative, periodontal, prosthodontic, and surgical planning.	The authors emphasize that close interdisciplinary planning improves esthetic, functional, skeletal, and periodontal outcomes in complex adult patients.	2 years
Digital orthodontic-restorative case report. Garcia-Torres et al. [32], 2024.	A patient with Class II occlusion and defective composite veneers was treated with orthodontics, digital occlusal analysis, diagnostic scans, digital wax-up/mock-up, and all-ceramic restorations.	The treatment improved occlusion, esthetics, and function, and the restorations remained stable with no wear.	3 years
Full-mouth rehabilitation case report. Kourtis [33], 2025.	An 18-year-old patient with amelogenesis imperfecta underwent second orthodontic treatment, conservative periodontal therapy, and full-mouth rehabilitation with all-ceramic restorations.	The patient was satisfied with the esthetic and functional outcome, and the clinical situation remained stable.	10 years
Interdisciplinary mutilated dentition case report. Liaw et al. [34], 2021.	A patient with multiple missing teeth, bite collapse, crossbites, deep bite, and skeletal discrepancy was treated with mini-implant anchorage, autotransplantation, one implant, and prosthetic rehabilitation.	Maxillary intrusion and mandibular counterclockwise rotation improved facial profile without orthognathic surgery, and autotransplantation reduced the need for multiple implants.	7 years and 8 months
Full-mouth rehabilitation clinical report. Cheong et al. [35], 2019.	A 20-year-old patient with collapsed occlusal plane, missing teeth, inadequate OVD, and esthetic/functional concerns was treated with periodontal therapy, orthodontics, endodontics, surgery, and prosthodontics.	The multidisciplinary sequence improved occlusal plane, tooth alignment, OVD, esthetics, and function.	Not reported
Adjunctive orthodontics and esthetic rehabilitation case report. Viet at al. [36], 2025.	A 29-year-old patient with Class III tendency, missing lateral incisors, spacing, and edge-to-edge occlusion was treated with fixed orthodontics, mini-screws, implant therapy, gingivoplasty, and veneers.	The interdisciplinary approach improved smile esthetics, occlusion, and facial appearance.	6 months
One-stage virtual planning case report. Nota et al. [37], 2022.	A complex orthodontic/prosthetic rehabilitation was planned using a one-stage virtual workflow combining gnathological, orthodontic, and prosthetic phases.	The one-stage virtual plan improved treatment visualization, reduced the risk of accumulated planning errors, and helped motivate the patient by previewing the expected result.	1 year
Preprosthetic orthodontic intervention clinical report. Bidra and Uribe [38], 2012.	A partially edentulous patient with generalized wear and malocclusion was treated with occlusograms, visualized treatment objectives, diagnostic waxing, TAD-assisted orthodontics, and prosthodontic rehabilitation.	The treatment restored esthetics and occlusion while preserving natural teeth, and the restorations and tooth positions remained stable.	2.5 years
Prosthetically guided orthodontics case series/report. Venezia et al. [39], 2022.	Three conformative rehabilitation cases were treated using prosthetically guided orthodontics with digital planning and clear aligner therapy before definitive prosthetic treatment.	The approach produced predictable esthetic results, high patient satisfaction, and minimally invasive prosthetic rehabilitations without negatively affecting function.	Not reported

## Data Availability

Data presented in this study are available on request from the corresponding author.
